# Weaning in early neurological-neurosurgical rehabilitation in Germany – results from a nationwide online survey

**DOI:** 10.3389/fneur.2025.1700482

**Published:** 2026-01-12

**Authors:** Oliver Summ, Heiko Frers, Andreas Klausen, Fabian Otto-Sobotka, Marcus Pohl, Rainer Röhrig, Tobias Schmidt-Wilcke, Kristin Schröder, Antje Timmer, Anette Weigel, Thomas Platz, Martin Groß

**Affiliations:** 1 Faculty of Medicine and Health Sciences, Carl von Ossietzky Universität Oldenburg, Oldenburg, Germany; 2 Evangelisches Krankenhaus Oldenburg, Oldenburg, Germany; 3 Klinikum Oldenburg, Oldenburg, Germany; 4 VAMED Klinik Schloss Pulsnitz, Pulsnitz, Germany; 5 Deutsche Gesellschaft für Neurorehabilitation, Bonn, Germany; 6 Institute of Medical Informatics, Universitätsklinikum Aachen, Aachen, Germany; 7 Bezirksklinikum Mainkofen, Deggendorf, Germany; 8 Jade Hochschule Oldenburg, Oldenburg, Germany; 9 Deutsche Gesellschaft für Atmungstherapie, Oldenburg, Germany; 10 BDH Klinik Greifswald, Greifswald, Germany; 11 Neurorehabilitation Research Group, Universitätsmedizin Greifswald, Greifswald, Germany; 12 MEDIAN Klinik Bad Tennstedt, Bad Tennstedt, Germany; 13 Deutsche Interdisziplinäre Gesellschaft für Außerklinische Beatmung und Intensivversorgung, Göttingen, Germany

**Keywords:** decannulation, early neurological-neurosurgical rehabilitation, mechanical ventilation, neurocritical care, neurointensive care, weaning

## Abstract

**Background:**

In Germany, approximately 1,100 beds are allocated to early neurological-neurosurgical rehabilitation (ENNR) for patients with severe neurological illness requiring weaning from mechanical ventilation. Specialized ENNR institutions play a major role in maintaining ICU capacity in acute care hospitals and in reducing the number of patients dependent on home intensive care. However, nationwide structural data on ENNR weaning centers–together with detailed patient characteristics–remain unavailable. This lack of information poses significant challenges for healthcare planning, resource allocation, and understanding the national weaning capacity.

**Methods:**

In June 2022, an online survey was conducted to collect structural data on institutions and wards, as well as cross-sectional clinical data from mechanically ventilated neurological patients. The survey was sent to the members of the weaning commission of the German Society for Neurological Rehabilitation (DGNR), to participants of a preceding study, and also to institutions recruited through personal communication.

**Findings:**

Data were collected from 24 institutions, including 46 wards and 182 patients. Institutions showed considerable variability in diagnostic and therapeutic resources; however, most relied on multidisciplinary teams and intensive monitoring, indicating high medical complexity of the patient population. The most frequent primary diagnoses included ischemic stroke (*n =* 27, 15%), intracerebral hemorrhage (*n =* 21, 12%), hypoxic–ischemic encephalopathy (*n =* 12, 7%), and subarachnoid hemorrhage (*n =* 12, 7%). The most frequent comorbidities were coronary heart disease (*n =* 40, 22%), left ventricular failure (37, 20%), and COPD (37, 20%). Mechanical ventilation was predominantly invasive (*n =* 180, 99%). The median number of days on mechanical ventilation during the stay in ENNR was 22 (IQR 9–41) days, and the median duration of ventilation was 18 (IQR 10–24) h during the last 24 h before data collection. Fourteen (8%) patients needed renal replacement therapy. Palliative therapy limitations were implemented in 22 (12%) patients.

**Interpretation:**

Institutions providing weaning in ENNR commonly treat older, medically complex patients who have undergone pretreatment in acute intensive care units. These patients typically present with severe neurological illness accompanied with cardiopulmonary comorbidities. In order to meet their needs, multidisciplinary teams deliver rehabilitation, intensive care, and palliative care.

## Introduction

Worldwide societies are confronted with an aging population and therefore a rising prevalence of neurological disease, multimorbidity, disability, and need for life-supporting technologies (1–10). Medical progress has improved survival in acute and chronic progressive neurological conditions, many of which go along with a continued need of medical care and nursing (11, 12). Over the last decades, medical progress in acute care has been paralleled by an evolution of care structures for patients with prolonged critical illness, including specialized weaning centers and rehabilitation facilities (13). In Germany, early neurological-neurosurgical rehabilitation (ENNR) was established during the 1990s for severely affected neurological patients. Multidisciplinary teams of specialized physicians, nurses, physiotherapists, speech and language therapists, occupational therapists, neuropsychologists, social workers, music therapists, and respiratory therapists treat patients to reduce disability and to optimize participation. Over the years, the majority of facilities providing ENNR have adapted life-sustaining therapies such as mechanical ventilation, kidney replacement, or left ventricular assist devices. A guideline on prolonged weaning in ENNR was first published in Germany in 2017 and updated in 2025 (14). In 2020, the German Society for Neurorehabilitation already identified 1,094 existing beds destined for prolonged weaning in ENNR (15) and began certifying ENNR weaning centers in 2021 (16). By June 2022, when data for the current study were collected, six centers had been certified. Data from the certification of the first 13 centers were published in February 2024 (17) and by July 2025, 30 centers were successfully certified or recertified. The German Society for Pulmonology and Mechanical Ventilation (DGP) and the German Society for Anaesthesiology and Intensive Care Medicine (DGAI) also certify institutions providing weaning from mechanical ventilation, but their centers are not required to provide rehabilitation capacities for patients successfully weaned. Owing to different definitions of “weaning” and variations in the diagnoses treated, outcomes such as weaning success and mortality are not comparable between neurological and pulmonological weaning centers (13, 18). In recent years, institutions specialized in weaning neurological patients are a vital component of German healthcare, positioned at the intersection of acute intensive care and subsequent rehabilitation, in-community care and home intensive care.

However, a cross-sectional national survey covering detailed data of individual institutions, wards, and mechanically ventilated patients is still lacking. Consequently, this study was designed to inform policymakers and healthcare providers and to prepare future longitudinal studies by answering the following research questions:

1. What are the features of institutions providing weaning in ENNR regarding the regional setting, their affiliation with acute care or rehabilitation hospitals, leadership, diagnostic and therapeutic methods, certifications, and treatment capacities?
2. What are the characteristics of wards providing weaning in ENNR regarding treatment capacities, and organization of multidiscilinary teamwork?
3. What are the patients’ characteristics regarding diagnoses, comorbidities, mechanical ventilation, monitoring, diagnostic procedures, life-supporting therapies, catheters, multidrug-resistant bacteria, and palliative treatment strategies?

## Methods

This study employed a descriptive, exploratory design using a non-probability convenience sample. Because this sampling strategy does not permit population-level representativeness, the findings should be interpreted as descriptive of the participating institutions rather than reflective of the full national healthcare landscape.

Survey items were developed through a structured review of existing ENNR guidelines, national certification criteria, and prior studies on neurological weaning centers (14, 17–23). The questionnaire comprised three sections—institution, ward, and patient level—mirroring the typical organizational structure of ENNR care. Content validity was established by eight members of the DGNR Weaning Commission, who reviewed all items in two iterative rounds and provided consensus-based revisions. Pretesting focused on clarity, relevance, and feasibility within the clinical workflow, and items were refined accordingly.

Consecutively, an online survey was conducted using SoSci Survey (version 3.2.44, SoSci Survey GmbH, Munich, Germany). The survey comprised three subsets of closed- and open-ended questions to collect data from institutions providing weaning in ENNR regarding characteristics of the institutions (Supplementary material 1), their wards for treatment of mechanically ventilated patients (Supplementary material 2), and currently treated patients meeting the inclusion criteria (Supplementary material 3). The survey was repeatedly pretested by the members of the weaning commission of the DGNR and by the authors involved in the conceptualization of the study to ensure clarity of the questions and the responses and to assess feasibility within the intended complex clinical environment. This was performed until all comments from the pretests were taken into account.

Wards providing weaning in ENNR to at least one patient were included, wards without mechanical ventilation on the day of data collection were excluded. All adult patients with continuous or intermittent mechanical ventilation on the day of data collection were included and patients aged less than 18 years were excluded.

Data on institutions included information on the geographic location, type and size of the facilities, the head of department, medical specialists, capacities for mechanical ventilation, certifications by medical associations, diagnostic methods, therapeutic procedures, out-of-hospital-ventilation related procedures, rehabilitative methods, and palliative care and ethics. Regarding wards, data were collected on size, conference frequencies and topics, and on the team involved in the conferences. Patient data comprised demographic aspects, diagnoses, pretreatment in other facilities, mechanical ventilation, other life supporting therapies, catheters, monitoring, and palliative restrictions of therapy.

A mailing list was built, including participants of a former study on mechanical ventilation in neurological organizational units in Germany, who had declared intent to participate in future studies, the weaning commission of the German Society for Neurological Rehabilitation (DGNR), and further institutions recruited by personal communication (18). The survey was sent to 48 institutions providing ENNR for mechanically ventilated patients in June 2022. An earlier survey using the mailing list of the DGNR found 68 of those institutions; however, the true number of institutions is not known. A coverage of approximately two-third of all institutions by the mailing list was assumed.

### Data analysis

Descriptive analysis was conducted by reporting absolute and relative frequencies for categorical variables and the median with interquartile range (IQR) for continuous metric variables. Missing data were accounted for by specifying the sample size (*n*) for each item.

### Registration and ethics

This study was registered in the German clinical trials register (Clinical trial identifier DRKS00019926; Federal Institute for Drugs and Medical Devices, Bonn, Germany) and approved by the Medical Ethics Committee of the University of Oldenburg, Niedersachsen (ref. 2019-151, Supplementary material 4). Secondary approvals were collected from the Medical Ethics Committees of the Medical University Hannover, Niedersachsen (ref. 9358_BO_K_2020), of the University of Rostock, Mecklenburg-Vorpommern (ref. A2020-0215), of the Medical Association of Baden Württemberg (ref. B-F-2020-126), of the Medical Association of Brandenburg [ref. AS83(bB)/2020], of the Medical Association of Nordrhein (ref. 2,020,441), of medical association Rheinland-Pfalz (ref. 2021-16,079), of the Medical Association of Sachsen (ref. EK-BR-113/20–1), and of the Medical Association of Hessen (ref. 2020-2143-zvBO). No secondary approvals were needed from the federal provinces of Berlin, Bavaria, and Schleswig-Holstein, as, according to the local medical professional code, the approval obtained by the Medical Ethics Committee of the University Oldenburg was regarded as sufficient. The study conforms with the principles of the World Medical Association Declaration of Helsinki (World Medical, 2013). Informed consent was not required according to the German data protection law—Datenschutzgrundverordnung, article 5 (1) b, article 89 (1), article 6 (4) a-e, article 9(2)j, and article 9 (3)—as only routine clinical data were collected, patient data were anonymized, no risks for the patients were expected, and the project was supervised by healthcare professionals.

## Results

### Institutions’ characteristics

Out of 68 institutions providing weaning in ENNR, which had been reported otherwise (15), 48 were included in the mailing list, and 24 participated in the study, resulting in a response rate of 50%. All 24 datasets were included in the analysis. Questions were answered by all participants, if not stated otherwise.

Fourteen (58%) of these institutions were in rural areas, four (17%) in cities of up to 100,000 inhabitants, three (13%) in cities of 100,000–500,000 inhabitants, and three (13%) in larger cities of >500,000 inhabitants. The institutions were part of rehabilitation hospitals (*n* = 18, 75%), specialized care hospitals (*n* = 3, 13%), maximum care hospitals (*n* = 2, 8%), and a university hospital (*n* = 1, 4%). In Germany, there are different levels of acute care hospitals: basic care, specialized care, maximum care, and university hospitals, of whch the last two provide the broadest spectrum of diagnostic and therapeutic methods. The size of those rehabilitation hospitals or acute care hospitals was reported to be ≤250 beds by 11 (46%), 251–500 beds by 9 (38%), 501–1,000 beds by 3 (13%), and >1,000 beds by 1 (4%). The total number of beds suitable for mechanically ventilated neurological patients was 605, and the median number per institution was 17 (IQR 10–28). The participating institutions provided various specialized diagnostic and therapeutic methods, but the penetration of those methods varied (Table 1). All institutions provided blood gas analysis, fiberoptic endoscopic evaluation of swallowing, bronchoscopy, neurological electrophysiology, and sonography. Approximately 21 (88%) institutions also provided computer tomography themselves, while 3 (13%) provided it in cooperation with other institutions. However, methods necessary for non-invasive out-of-hospital ventilation, such as polygraphy (*n* = 8, 33%) and transcutaneous capnometry (*n* = 12, 50%), and extracting data from out-of-hospital ventilators (*n* = 9, 38%) were less prevalent.

**Table 1:** Characteristics of departments providing weaning in early neurological-neurosurgical rehabilitation, institutions, and certifications.

Institutions		
Category	Specification	Number
Diagnostic methods	Polygraphy	8 (33%)
	Spirometry	17 (71%)
	Blood gas analysis	24 (100%)
	End-exspiratory capnometry	23 (96%)
	Transcutaneous capnometry	12 (50%)
	Fiberoptic endoscopic evaluation of swallowing	24 (100%)
	Bronchoscopy	24 (100%)
	Gastroscopy	17 (71%)
	CT^1^	21 (88%)
	MRI^2^	10 (42%)
	Neurological electrophysiology	24 (100%)
	Sonography	24 (100%)
	Emergency laboratory	23 (96%)
	CSF^3^ diagnostics	15 (63%)
Rehabilitative methods	Gaze-controlled communication systems	15 (63%)
	*Speech-*generating devices	13 (54%)
	Counseling for relatives	24 (100%)
	Group counseling for relatives	7 (29%)
Palliative care and ethics facilities	Palliative care team	18 (75%)
	Palliative care consultation	8 (33%)
	Palliative care ward	4 (17%)
	Ethics committee	22 (92%)

Departments		
Category	Specification	Number
Head of weaning department (medical specialty)	Neurologist	11 (46%)
	Anesthesiologist	8 (33%)
	Pulmonologist	2 (8%)
	Other specialty or cooperative leadership	3 (13%)
Therapeutic spectrum for mechanically ventilated patients	Invasive ventilation	24 (100%)
	Noninvasive ventilation	21 (88%)
	Mechanical insufflation-exsufflation	21 (88%)
	Kidney replacement	7 (29%)
	LVAD^4^	12 (50%)
Out-of-hospital ventilation	Initiation of OV^5^	22 (92%)
	Control of OV^5^ in an inpatient setting	18 (75%)
	Control of OV^5^ in an outpatient setting	5 (21%)
	Emergency admission of patients with OV^5^	7 (29%)
	Extracting data from out-of-hospital ventilators	9 (38%)

Certifications		
Category	Specification	Number
Certifications by medical associations	Weaning center, DGP^6^	2 (8%)
	Center for weaning in early neurological-neurosurgical rehabilitation (ENNR), DGNR^7^, certified	3 (13%)
	Center for weaning in ENNR, DGNR^7^, applied for certification	2 (8%)
	Weaning from mechanical ventilation, DGAI^8^	1 (4%)
	Center for spinal cord injury, DMGP^9^	3 (13%)
	Sleep laboratory^10^	2 (8%)
Total		24

^1^Computed tomography. ^2^Magnetic resonance imaging. ^3^Cerebrospinal fluid. ^4^Left ventricular assist device. ^5^Out-of-hospital ventilation. ^6^German Respiratory Society. ^7^German Society for Neurorehabilitation. ^8^German Society for Anesthesiology and Intensive Care Medicine. ^9^German Speaking Society for Spinal Cord Injury. ^10^German Society for Sleep Research and Medicine.

### Wards

Forty-six wards with the capability to provide ENNR to mechanically ventilated patients with a median number of ventilation beds of 9 (IQR 5–20) participated in this study. Information on the frequency of multiprofessional team conferences to be held on the goals of treatment for each individual patient, the participating professions, and the selected topics of the conferences were provided by 37 wards. Team conferences were held in 21 (57%) wards once per week, every day, respectively, every working day in 10 (27%), twice per week in 4 (11%), three times per week in 1 (3%) and not at all in 1 (3%). Respiratory therapy or weaning from the respirator were reported to be conference topics in 35 (95%) wards, while ethics committee involvement or palliative care were reported to be topics of the conferences in 27 (72%) wards. Medical specialists–predominantly neurologists, anesthesiologists, and internal specialists–always took part in the team conferences. Each of the following disciplines attended in more than two third of the wards: nurses, physiotherapists, occupational therapists, speech and language therapists, (neuro)psychologists, and respiratory therapists (Table 2).

**Table 2:** Participation of medical and non-medical disciplines in the multidisciplinary conferences.

Category	Specification	Number of wards
Medical disciplines	Neurologist	30 (81%)
	Anesthesiologist	17 (46%)
	Internal specialist	16 (43%)
	Neurosurgeon	2 (5%)
	General practitioner	1 (3%)
	Specialist registrar	27 (73%)
Non-medical disciplines	Nurse	36 (99%)
	Respiratory therapist^1^	26 (70%)
	Speech and language therapist	32 (86%)
	Physiotherapist	35 (95%)
	Occupational therapist	31 (84%)
	(Neuro-)Psychologist	28 (76%)
	Music therapist	11 (30%)
	Art therapist	1 (3%)
	Physician assistant	3 (8%)
	Educationalist	4 (11%)
Total		37

^1^Qualified or in training.

### Patients’ characteristics

A total of 128 (70%) male and 54 (30%) female patients were included. The median age was 68 years. Thirty-eight (21%) of the patients had a body weight of 100 kg or more. Patients being transferred from other hospitals (*n =* 153, 84%) were done so by the following medical specialties: anesthesiologic and interdisciplinary intensive care (*n =* 51, 33%), neurosurgery (*n =* 38, 25%), internal medicine (*n =* 35, 23%), neurology (*n =* 18, 12%), heart surgery (*n =* 8, 5%) and other specialties (*n =* 3, 2%), and most of them had been treated on intensive care units of the assigning hospital (*n =* 147, 96%). The median length of stay in the transferring acute care hospital was 23 days (IQR 16–32). The most frequent primary diagnoses leading to acute hospital treatment were ischemic stroke (*n =* 27, 15%), intracerebral hemorrhage (*n =* 21, 12%), hypoxic–ischemic encephalopathy (*n =* 12, 7%), subarachnoid hemorrhage (*n =* 12, 7%), intracranial tumor (*n =* 8, 4%) and sepsis (*n =* 7, 4%) (Table 3). Of all diagnostic methods, patients most often required EEG (*n =* 83, 46%), computer tomography (*n =* 79, 43%), bronchoscopy (68, 37%), and FEES (*n =* 70, 28%). Fourteen patients (8%) required intermittent or continuous kidney replacement. Invasive blood pressure monitoring took place in 121 (67%). A central venous catheter was present in 103 (57%), a nasogastric tube in 107 (59%), a percutaneous gastrostomy or jejunostomy tube in 65 (36%), and a transurethral urinary catheter in 170 (94%) (Table 4). For all 182 patients, information was available on the duration of ventilation during the last 24 h before data collection (median 18 h, IQR 10–24 h) and on the number of days on mechanical ventilation during the stay in ENNR (median 22 days, IQR 9–41 days). Mechanical ventilation was predominantly invasive (*n =* 180, 99%) (Table 5).

**Table 3:** Primary diagnoses leading to treatment in acute care hospitals, neurological complications of ICU treatment, and conditions at the time of data collection.

Category	Specification	Number
Primary diagnoses leading to treatment in the acute care hospital	Ischemic stroke	27 (15%)
	Intracerebral hemorrhage	21 (12%)
	Hypoxic–ischemic encephalopathy	12 (7%)
	Subarachnoid hemorrhage	12 (7%)
	Sepsis	10 (5%)
	Intracranial tumor	8 (4%)
	Craniocerebral trauma	7 (4%)
	Subdural hematoma	5 (3%)
	Polytrauma	5 (3%)
	Amyotrophic lateral sclerosis	4 (2%)
	Myasthenia Gravis	4 (2%)
	CNS infection	4 (2%)
	COVID-19 infection	4 (2%)
	Abdominal diseases	4 (2%)
	Status epilepticus	3 (2%)
	Guillain-Barré-syndrome	3 (2%)
	Autoimmune encephalitis	3 (2%)
	Heart failure	3 (2%)
	Other Trauma	3 (2%)
	Pneumonia	3 (2%)
	Complicated cardiac surgery	3 (2%)
	Intoxication	2 (1%)
	Other/unclassified diagnoses	32 (18%)
Neurological complications of ICU treatment	Ischemic stroke	33 (18%)
	CIP/CIM^1^	27 (15%)
Conditions at the time of data collection	Coronary heart disease	40 (22%)
	Left ventricular failure	37 (20%)
	COPD	37 (20%)
	Kidney failure	25 (14%)
	Liver failure	9 (5%)
Total		182

^1^Critical-illness polyneuropathy/myopathy.

**Table 4:** Diagnostic examinations during stay in ENNR, life supporting technologies during the last 24 h before data collection, as well as monitoring, catheters, and hygiene isolation during data collection.

Category	Specification	Number of patients
Diagnostic examinations	Transcutaneous capnometry	18 (10%)
	Spirometry	20 (11%)
	Polygraphy	8 (4%)
	Bronchoscopy	68 (37%)
	FEES^1^	70 (28%)
	Cranial MRI^2^	17 (9%)
	Cranial CT^3^	79 (43%)
	Thoracic CT^3^	19 (10%)
	Abdominal CT^3^	12 (7%)
	Other CT^3^	8 (4%)
	EMG/NCV^4^	30 (16%)
	Evoked potentials	26 (14%)
	EEG^5^	83 (46%)
	CSF^6^ diagnostics	19 (10%)
	*Patients with available data*	*n = 182*
Life-supporting technologies	Continuous kidney replacement	3 (2%)
	Intermittent kidney replacement	11 (6%)
	LVAD^7^	1 (1%)
	*Patients with available data*	*n = 181*
Monitoring	ECG^8^	175 (97%)
	Invasive blood pressure	121 (67%)
	End-tidal capnometry	92 (51%)
	BGA^9^	174 (96%)
	Continuous EEG	1 (1%)
	ICP^10^	6 (3%)
	Cardiac output monitoring	0 (0%)
	*Patients with available data*	*n = 181*
Catheters	Thoracic drain	5 (3%)
	Central venous catheter	103 (57%)
	Ventricular CSF^6^ drain	10 (6%)
	Lumbar CSF^6^ drain	0 (0%)
	Nasogastric tube	107 (59%)
	Percutaneous gastrostomy or jejunostomy tube	65 (36%)
	Transurethral urinary catheter	170 (94%)
	Suprapubic urinary catheter	3 (2%)
	*Patients with available data*	*n = 181*
Hygiene isolation	Prophylactic isolation	71 (39%)
	Colonization/infection with 3-MRGN	15 (8%)
	Colonization/infection with 4-MRGN	5 (3%)
	Colonization/infection with MRSA	4 (2%)
	Colonization/infection with VRE	12 (7%)
	Colonization/infection with *Acinetobacter baumanii*	1 (1%)
	Infection with *Clostridium difficile*	1 (1%)
	Local proceedings regarding COVID-19	6 (3%)
	*Patients with available data*	*n = 182*

^1^Flexible endoscopic evaluation of swallowing. ^2^Magnetic resonance imaging. ^3^Computed tomography. ^4^Electromyography/nerve conduction velocity. ^5^Electroencephalography. ^6^Cerebrospinal fluid. ^7^Left ventricular assist device. ^8^Electrocardiography. ^9^Blood gas analysis. ^10^Intracranial pressure.

**Table 5:** Technical aspects of mechanical ventilation.

Category	Specification	Value	Data available from *n* patients
Interface	Endotracheal tube	15 (8%)	182
	Tracheal cannula	165 (91%)	
	Mask	2 (1%)	
Type of respirator	Intensive care ventilator	173 (95%)	
	Home ventilator	9 (5%)	
Ventilation mode	Pressure controlled/BiPAP	59 (32%)	
	Pressure assisted	116 (64%)	
	Other or combination	7 (4%)	
PEEP (cmH_2_O)		Median 11 (IQR 10–13)^1^	182
FiO_2_ (%)		Median 30 (IQR 30–35)^2^	165
Mechanical insufflation-exsufflation		23 (13%)	181

Palliative limitations of therapy had been applied to 22 (12%) patients. These limitations were applied because of patient’s preferences reported by representatives in 10 (5%) or documented in advance directives in 5 (3%), lack of medical indication in 4 (2%), and verbally expressed patient’s preferences in 3 (2%). Specific limitations of therapy were as follows: no CPR (*n =* 20, 11%), no kidney replacement therapy (*n =* 10, 5%), no escalation of therapy (*n =* 7, 4%), no catecholamine treatment (*n =* 5, 3%), no ICU treatment (*n =* 4, 2%), no antibiotic treatment (*n =* 2, 1%), and no enteral or parenteral feeding (*n =* 1, 1%).

## Discussion

This study extends on previous research by complementing existing data on the characteristics of institutions and wards providing weaning in ENNR, as well as on mechanically ventilated neurological and neurosurgical patients treated in these settings (17–23). The participating institutions represent more than 50% of the total capacity available for weaning in neurological-neurosurgical early rehabilitation in Germany (15). Given the complex design of the survey with three interconnected and detailed datasets, the response rate of 50% can be considered as high.

Notably, although 605 beds were designed as suitable for mechanical ventilation, only 182 patients with mechanical ventilation were included in this study. This is probably due to several factors: Monitoring wards in ENNR not only accommodate patients with mechanical ventilation but also patients with tracheal tubes and other conditions requiring vital parameter monitoring, as well as patients waiting for beds on non-monitoring wards. In addition, staff shortage may have further limited treatment capacities.

### Institutions

Structural requirements for institutions providing weaning in ENNR are determined by the patients´characterstics: The spectrum of underlying diseases is broad, and severe comorbidities are frequently present. Patients often suffer from complex, disabling, and life-threatening symptoms. These include impairment of cough, swallowing and breathing; disorders of consciousness; vegetative symptoms; paralysis; spasticity; impaired perception or cognitive function; depression; anxiety; and pain (14, 24, 25). Consequently, several medical specialists from different conservative and surgical disciplines are involved in the institutions (14).

Institutions specialized in weaning in ENNR provided a wide range of diagnostic and therapeutic methods; however, the penetration of these methods varies. This fact is already addressed by the certification of centers for weaning in ENNR by the DGNR (16). The need for rehabilitation technologies becomes evident through the fact that most centers have established sophisticated technical solutions for augmentative and alternative communication (AAC) as gaze-controlled communication devices and speech-generating devices (Table 1). Generally, a family-oriented therapeutic approach is present, reflected by the fact that counseling for relatives is provided in all institutions. The penetration of various methods required for out-of-hospital ventilation, such as non-invasive ventilation, polygraphy, spirometry, transcutaneous capnometry, mechanical insufflation-exsufflation, and extraction of data from out-of-hospital ventilators, is particularly incomplete (26). This aligns with findings from an earlier study (19).

### Wards

Multidisciplinary teamwork is organized through regular physician-led team conferences, which were established in all but one of the 37 wards with available data on conferences. Medical specialists from different disciplines–predominantly neurologists, anesthesiologists, and internal specialists–nurses, physiotherapists, occupational therapists, speech and language therapists, (neuro)psychologists, and respiratory therapists may be regarded as the core team on weaning wards in ENNR. The composition of the multidisciplinary teams in ENNR is partially predetermined by the German regulatory framework, which mandates an average of 300 min of therapy per patient per day. These 300 min comprise therapeutic nursing, physiotherapy, physical therapy, occupational therapy, (neuro-) psychology, speech- and language therapy, and music therapy. In contrast, other disciplines are less likely to be integrated in the rehabilitation process. The regulatory framework formally will include the respiratory therapist from 2026.

### Patients

Most of the patients were older than 65 years. Thus, according to the most common definition in industrial countries, these patients may be classified as older adults (27). A recent German register study of more than 11,000 patients documented that higher age is significantly associated with weaning failure and death in the weaning unit (13). The fact that most patients were referred from ICUs explains why typical aspects of ICU treatment had been present in the last 24 h before data collection in most patients: invasive ventilation, invasive blood pressure monitoring, and central venous catheters. The spectrum of diagnostics performed during the stay in ENNR was broad, with computed tomography, bronchoscopy, FEES, and EEG being the most frequently utilized.

The primary diagnoses leading to hospitalization–ischemic stroke, intracerebral hemorrhage, hypoxic ischemic encephalopathy, and subarachnoid hemorrhage being the most frequent–are consistent with typical admission profiles for Neuro-ICU and align with previous reports from institutions providing weaning in ENNR (28, 29). COPD, left ventricular failure, coronary heart disease, and kidney failure–are all associated with elevated ICU mortality (30–32). Notably, the proportion of patients with critical-illness-polyneuropathy/−myopathy (CIP/CIM) at the time of data collection was 15%. CIP/CIM is a typical complication of any critical illness, and patients with CIP/CIM may therefore be referred from any medical discipline to receive weaning in ENNR (33).

The median number of days on mechanical ventilation during the stay in ENNR (22 days, IQR 9–41 days) is consistent with is consistent with common definitions of prolonged weaning (34). A median PEEP of 11 cmH2O (IQR 10–13 cmH2O) was probably used because of pulmonary impairment. Dysphagia (35, 36) and cough insufficiency are typical for critically ill neurological patients in general, and COPD as a comorbidity was frequently observed in the study patients. While tracheostomy is a routine procedure in prolonged weaning, the aforementioned factors may moreover contribute to the ratio of invasive to non-invasive ventilation being as high as 90:1 among the patients (37).

Nearly 12% of the patients have palliative therapy restrictions. Most institutions meet the palliative care needs of the patients with a palliative care team and an ethics committee. The palliative care needs of patients in ENNR are increasingly recognized (38). Mechanically ventilated patients in ENNR require a holistic treatment concept comprising rehabilitation, intensive care, and palliative care.

### Outcomes and treatment capacities

The paramount treatment goals in ENNR are weaning patients from mechanical ventilation and subsequent decannulation. Current data indicate that 75% (20) of patients treated in ENNR can be successfully weaned from mechanical ventilation, and 57% (21) of those weaned may undergo decannulation in the further process. Earlier studies have reported weaning rates of 92% (39), 68% (40), 70% (22), 82% (41), and 65% (23) in patients in ENNR. One study showed that 43% of patients with CIP and 26% of patients with cerebrovascular disease who had been successfully weaned from mechanical ventilation in ENNR achieved the ability to live independently at home (42). However, those who cannot be weaned from the ventilator and decannulated remain dependent on home intensive care and multidisciplinary treatment (43).

### Considerations for implementation on an international level and research needs

Respiratory impairment in severely ill neurological and neurosurgical patients may arise from dysphagia, cough insufficiency, ventilatory pump failure, acquired hypoventilation syndrome, respiratory pattern disorders, and comorbidities (44). Consequently, many of these patients require tracheostomy or mechanical ventilation. The necessity of multidisciplinary rehabilitation for ventilator-dependent patients has already been recognized by neurologists, pulmonologists, and spinal cord injury specialists (16, 45–47). Over the past two decades, the literature on early rehabilitation for patients with mechanical ventilation has grown substantially (48). However, definitions of “early rehabilitation” vary considerably. A recent meta-analysis proposed the following definition: “rehabilitation within ≤48 or ≤72 h after ICU admission or mechanical ventilation” (49). Studies in this field also examined diverse interventions, most frequently in the ICU setting (48, 50, 51). In Germany, several multicenter studies have investigated weaning from mechanical ventilation in the ENNR setting (17–23). In contrast, publications from other countries describing institutionalized models of multidisciplinary rehabilitation for mechanically ventilated patients are typically single-center data and do not focus on the broader population of neurological and neurosurgical patients (46, 52).

The detailed institutional and patient characteristics presented in this study underscore the structural and multidisciplinary requirements of ENNR weaning care and may support ongoing discussions regarding healthcare planning, certification standards, and future research priorities. However, there remains insufficient evidence to guide the optimal design of multidisciplinary interventions for neurological and neurosurgical patients.

Further research is required to systematically investigate healthcare structures providing weaning and neurorehabilitation on an international level. Additionally, studies should aim to generate deeper insight into the patient journey of critically ill neurological patients from intensive care via rehabilitation to subsequent community re-integration. Finally, a patient registry is needed to identify factors influencing key outcomes, including complications, weaning and decannulation rate, and care settings after discharge.

## Limitations

The complete population of neurological and neurosurgical patients requiring weaning in Germany is not known; therefore, it remains unclear which proportion of the complete population was included in this study. In addition, there is a possible selection bias driven by a positive attitude of the participating institution toward research: patient characteristics may differ from the overall population and structural characteristics may vary when compared with institutions with a more reserved attitude toward study participation. Hence, heterogeneity among centers might be underreported by the data presented. The objective of this study was to describe the current situation in Germany. The healthcare landscape in other countries may be substantially different. Data on the delivery of care by non-medical disciplines per individual patient and treatment outcomes were not collected. Therefore comparability with other models of care is limited. Finally, no formal psychometric validation (e.g., test–retest reliability, internal consistency, or construct validity) was performed. The questionnaire should therefore be interpreted as content-validated, without psychometrical validation.

## Conclusion

Institutions providing weaning in ENNR commonly treat elderly patients with high medical complexity, most of whom have undergone treatments in acute intensive care units. The patients typically present with severe neurological illness together with cardiopulmonary comorbidities. To address these complex medical needs, multidisciplinary teams deliver rehabilitation, intensive care, and palliative care also.

## Data Availability

The datasets presented in this study can be found in online repositories. The names of the repository/repositories and accession number(s) can be found at: DOI: 10.17632/9t5r6r6ppj.1 (https://data.mendeley.com/datasets/9t5r6r6ppj/1).
