# Supplementary material for: Weaning in early neurological-neurosurgical rehabilitation in Germany – results from a nationwide online survey
Source: Front Neurol. 2026 Jan 12;16:1700482. doi: 10.3389/fneur.2025.1700482 (PMC12832523; doi:10.3389/fneur.2025.1700482)
Supplement: Supplementary file 1 [file Data_Sheet_1.pdf]

CARL VON OSSIETZKY universität OLDENBURG
FAKULTÄT VI
MEDIZIN UND GESUNDHEITSWISSENSCHAFTEN

CARL VON OSSIETZKY UNIVERSITÄT OLDENBURG · 26111 OLDENBURG

Evangelisches Krankenhaus Oldenburg
Klinik für Neurologische Intensivmedizin und Frührehabilitation
Dr. Martin Groß
Steinstr. 13-17
26122 Oldenburg

Medizinische Ethikkommission
-Geschäftsstelle-

TELEFONDURCHWAHL
+49 (0)441 798 31 09
FAX
+49 (0)441 798 4745
E-MAIL
med.ethikkommission@uni-oldenburg.de

VORSITZENDER
Prof. Dr. Frank Griesinger

JURISTIN
Ass. jur. Carola Alvarez Castillo

Unser Zeichen: 2019-151 (bitte stets angeben)

Antragsteller: Dr. Martin Groß

Titel: Neurologische Beatmungsmedizin in Deutschland – NurBeat

Antrag vom: 16.12.2019, eingegangen am 20.12.2019

OLDENBURG, 14.01.2020

Freiwillige Beratung

Sehr geehrter Herr Dr. Groß,

Ihr Antrag hat mir als Vorsitzender der medizinischen Ethikkommission zur berufsrechtlichen Beratung vorgelegen.

Nach § 4 Absatz VI unserer Verfahrensregelung ist der Vorsitzende ermächtigt, zur Beschleunigung und Vereinfachung des Verfahrens im Einvernehmen mit einem Mitglied oder mehreren unter Einbeziehung der Geschäftsstelle zu entscheiden. Eine Pflichtberatung gem. § 15 BO ÄKN liegt hier nicht vor, so dass im verkürzten Verfahren entschieden werden kann.

Die medizinische Ethikkommission hat keine Bedenken gegen die Durchführung der Studie.

Sie empfiehlt,

- das Studienprotokoll zu versionieren, datieren und paginieren;
- Funktions-E-Mail-Adressen angeben zu lassen, sollten die teilnehmenden Einrichtungen am Benchmarking teilnehmen wollen.

ems
european medical school
oldenburg-groningen

POSTANSCHRIFT
D-26111 Oldenburg

PAKETANSCHRIFT
Ammerländer Heerstraße 114 - 118
D-26129 Oldenburg

BESUCHERANSCHRIFT
Ammerländer Heerstr. 140
D-26129 Oldenburg
V04-1-136/137

INTERNET
www.uni-oldenburg.de

BANKVERBINDUNG
Landessparkasse zu Oldenburg
IBAN DE46 2805 0100 0001 9881 12
BIC SLZODE22

Bitte beachten Sie noch folgende Punkte:

- Die Ethikkommission erwartet, dass ihr ohne Aufforderung ein Abschlussbericht mit dem beigelegten **Formular B** übermittelt wird.
- Unabhängig davon ist die Ethikkommission unaufgefordert und zeitnah über alle Änderungen am Prüfplan sowie den in diesem Antrag vorgelegten Dokumenten unaufgefordert und unverzüglich zu unterrichten. Ihr sind unaufgefordert alle schweren unerwünschten Ereignisse mitzuteilen, soweit sie im Zuständigkeitsbereich der Ethikkommission aufgetreten sind.
- Die ethische medizinische und juristische Verantwortung des Studienleiters und des an der Studie beteiligten medizinischen und wissenschaftlichen Personal bleibt entsprechend der Beratungsfunktion der medizinischen Ethikkommission durch diese Stellungnahme unberührt.
- Die Ethikkommission kann dieses Votum jederzeit zurückziehen oder ändern. Dies wird dem Antragsteller mitgeteilt.
- Bitte machen Sie dieses Votum und die der Begutachtung zugrundeliegenden Dokumente allen beteiligten Ärztinnen und Ärzten und Wissenschaftlerinnen und Wissenschaftlern zugänglich.

An der Beratung und Beschlussfassung haben keine Kommissionsmitglieder teilgenommen, die selbst an dem Forschungsvorhaben mitwirken oder deren Interessen davon berührt werden.

Wir bitten um Mitteilung der teilnehmenden Ärztinnen und Ärzte im Zuständigkeitsbereich der Universität Oldenburg, sobald diese bekannt sind bzw. sofern im Verlauf weitere Ärztinnen und Ärzte hinzukommen.

Wir möchten darauf hinweisen, dass die Stellungnahme der medizinischen Ethikkommission und die studienrelevante Korrespondenz an alle teilnehmenden Ärztinnen und Ärzte weiterzuleiten ist.

Bitte informieren Sie die Ethikkommission unter Nutzung des beigefügten Formulars A über den Beginn der Rekrutierung an Ihrem Studienzentrum.

Wir wünschen Ihnen bei der Durchführung Ihrer Studie viel Erfolg.

Mit freundlichen Grüßen

Prof. Frank Griesinger
Vorsitzender der medizinischen Ethikkommission

Eingereichte Unterlagen:
Anschreiben vom 16.12.2019
Formaler Antrag vom 18.12.2019
Studienprotokoll
Datenschutzrechtliche Unbedenklichkeitserklärung, E-Mail vom 01.11.2019
