# Supplementary material for: Weaning in early neurological-neurosurgical rehabilitation in Germany – results from a nationwide online survey
Source: Front Neurol. 2026 Jan 12;16:1700482. doi: 10.3389/fneur.2025.1700482 (PMC12832523; doi:10.3389/fneur.2025.1700482)
Supplement: Supplementary file 2 [file Data_Sheet_2.pdf]

Seite 01

INFO

Liebe Kolleginnen und Kollegen,

ich bedanke mich bei Ihnen, dass Sie Interesse an einer Teilnahme an der NurBeat-Studie haben und diese trotz allen Schwierigkeiten der letzten zwei Jahre mit Ihren Ethikanträgen und konstruktiven Rückmeldungen unterstützt haben. Mein besonderer Dank gilt auch dem Präsidium der Deutschen Gesellschaft für Neurorehabilitation, das durch die Übernahme der Schirmherrschaft für NurBeat die Umsetzung der Studie erst möglich gemacht hat. Wir hoffen, mit NurBeat wichtige inhaltliche Informationen für die weitere Ausgestaltung der Zertifizierung von Zentren für Beatmungsentwöhnung in der Neurologisch-neurochirurgischen Frührehabilitation und für den Aufbau eines Patientenregisters zu gewinnen. Wir hoffen auch, mit den Ergebnissen dieser Studie nachzuweisen, dass unsere Zentren, die Beatmungsentwöhnung und Rehabilitation verbinden, ein ebenso effizienter wie unverzichtbarer Bestandteil der Versorgungslandschaft in Deutschland geworden sind.

Seite 02

Hinweise zum Bearbeiten

Der Fragebogen setzt sich aus drei Teilen zusammen:

1. Allgemeine Daten zu Ihrer Einrichtung

Dieser Teil fragt allgemeine Daten Ihrer Einrichtung ab.
Das Bearbeiten dieses Teil dauert **etwa 10 Minuten.**

2. Fragen zu Ihren Stationen

Im allgemeinen Teil werden Sie gebeten, die Stationen zu nennen, auf den neurologische Beatmungspatienten behandelt werden.
Im zweiten Teil des Fragebogens bitten wir Sie, Angaben zu den von Ihnen genannten Stationen zu machen.
Das Bearbeiten dieses Teils des Fragebogens dauert **max. 10 Minuten** für jede Station.

3. Frage der Patientendaten

In diesem Teil möchten wir Sie bitten, Fragen zu jedem Patienten, der aktuell auf Ihren Stationen behandelt wird, zu beantworten.
Für jeden Patienten dauert die Eingabe der Daten ca. 15 Minuten.

Seite 03

Seite 04

Start AD

Bitte geben Sie die allgemeinen Daten zu Ihrer Einrichtung ein

Bevor Sie mit dem Ausfüllen des Fragebogens mit den Fragen zu den einzelnen Stationen und der Beantwortung der Fragen zu Ihren Patienten beginnen können, möchten wir Sie bitten, allgemeine Fragen zu Ihrer Einrichtung zu beantworten.

Das Beantworten dieser Fragen ist notwendig, da durch Ihre Antworten der weitere Ablauf des Fragebogens gestaltet wird.

Diese Fragen müssen nur einmal beantwortet werden!

Seite 05

Möchten Sie an einem Benchmarking teilnehmen, in dem Ihre Ergebnisse mit den anonymisierten Ergebnissen der anderen teilnehmenden Einrichtungen verglichen werden, dann wählen Sie bitte „Ja“ und geben Sie ihre Email-Adresse ein.

- ◯ ja [Bitte geben Sie Ihre Email-Adresse ein.]
- ◯ nein

Seite 06

jump5

In welchem Bundesland liegt Ihre Einrichtung?

[Bitte auswählen] ⌄

In welcher Region liegt Ihre Einrichtung?

- ◯ Ballungszentrum (Städte oder Stadtverbund > 500 000 Einwohner)
- ◯ Großstadt (100 000 bis 500 000 Einwohner)
- ◯ Stadtgebiet (bis 100 000 Einwohner)
- ◯ Ländliche Region

Handelt es sich bei Ihrer Einrichtung um eine Universitätsklinik, ein Akutkrankenhaus oder eine Rehabilitationseinrichtung?

- ◯ Universitätsklinik
- ◯ Akutkrankenhaus der Maximalversorgung
- ◯ Akutkrankenhaus der Schwerpunktversorgung
- ◯ Akutkrankenhaus der Grundversorgung
- ◯ Rehabilitationseinrichtung mit Versorgung der beatmeten Patienten im Krankenhausbereich (sogenannte §108-Betten nach SGB V; im manchen Bundesländer als Fachkrankenhaus mit limitiertem Versorgungsauftrag geführt)
- ◯ Rehabilitationseinrichtung mit Versorgung der beatmeten Patienten im Rehabilitationsbereich (sogenannte §111-Betten nach SGB V)
- ◯ Andere [Bitte beschreiben Sie Ihre Einrichtung]

Wie hoch ist die Anzahl der Betten in Ihrer Einrichtung?

- ○ weniger oder gleich 250 Betten
- ○ 251 bis 500 Betten
- ○ 501 bis 1000 Betten
- ○ mehr als 1000 Betten

Ist Ihre Einrichtung wie folgt zertifiziert/akkreditiert, ist eine Zertifizierung geplant oder beantragt?

	Zertifikat / Zentrumsanerkennung	Aktuell beantragt	Antrag im nächsten Jahr geplant geplant	Aktuell keine Planung	keine Angabe
Weaningzentrum (DGP)	○	○	○	○	○
Entwöhnung von der Beatmung (DGAI)	○	○	○	○	○
Querschnittzentrum (DMGP)	○	○	○	○	○
Schlaflabor (DGSM)	○	○	○	○	○
Streben Sie die Zertifizierung als „Zentrum für Beatmungsentwöhnung in der Neurologisch-neurochirurgischen Frührehabilitation“ an?	○	○	○	○	○

Welche der folgenden Behandlungen können in Ihrer Einrichtung durchgeführt werden?

- ☐ Nichtinvasive Beatmung
- ☐ Invasive Beatmung
- ☐ Sekretmanagement mit Mechanischem Insufflator-Exsufflator
- ☐ Nierenersatzverfahren
- ☐ Behandlung von Patienten mit Left ventricular Assist Device (LVAD„ Kunstherz“)
- ☐ Leberdialyse (MARS)
- ☐ Behandlung querschnittgelähmter Patienten
- ☐ Sonstiges: Bitte geben Sie weitere Therapiemöglichkeiten an

Seite 10

Welche Maßnahmen bezüglich außerklinisch beatmeter Patienten können in Ihrer Einrichtung durchgeführt werden?

- ☐ Einstellung einer außerklinischen Beatmung
- ☐ Stationäre Wiederaufnahme zur Kontrolle
- ☐ Ambulante Kontrolle
- ☐ Notfallmäßige stationäre Aufnahme außerklinisch beatmeter Patienten
- ☐ Auslesen von Heimbeatmungsgerätedaten
- ☐ Sonstiges: Bitte geben Sie weitere Maßnahmen an.

Seite 11

Welche Palliative Care-Dienste bietet Ihre Einrichtung an?

- ☐ Palliativteam vorhanden
- ☐ Palliativdienst/Palliativ-Konsiliardienst vorhanden
- ☐ Palliativstation vorhanden
- ☐ Abrechnung von OPS 8-982 (Palliativmedizinische Komplexbehandlung)
- ☐ Abrechnung von OPS 8-98e (Spezialisierte stationäre palliativmedizinische Komplexbehandlung)
- ☐ Abrechnung von OPS 8-98h (Spezialisierte palliativmedizinische Komplexbehandlung durch einen Palliativdienst)
- ☐ Klinisches Ethikkommittee vorhanden

question('AD12', '1-9')

Bitte geben Sie an, welche diagnostischen Möglichkeiten in Ihrer Einrichtung vorhanden sind, welche nicht und welche ggf. über eine zeitnahe Kooperation zur Verfügung stehen.

	vorhanden	zeitnah über Kooperation	nicht vorhanden
Polysomnographie	○	○	○
Polygraphie	○	○	○
Spirometrie	○	○	○
Blutgasanalyse	○	○	○
Endexspiratorische Kapnometrie	○	○	○
Transkutane Kapnometrie	○	○	○
Fiberendoskopische Schluckdiagnostik	○	○	○
Bronchoskopie	○	○	○
Gastroskopie	○	○	○

question('AD12', '10-17')

Bitte geben Sie an, welche diagnostischen Möglichkeiten in Ihrer Einrichtung vorhanden sind, welche nicht und welche ggf. über eine zeitnahe Kooperation zur Verfügung stehen.

	vorhanden	zeitnah über Kooperation	nicht vorhanden
Computertomographie	○	○	○
Magnetresonanztomographie	○	○	○
Neurologische Elektrophysiologie	○	○	○
Sonographie	○	○	○
Notfalllabor	○	○	○
Liquordiagnostik	○	○	○

Gibt es in Ihrer Einrichtung die Möglichkeit zur elektronisch unterstützten Kommunikation? Wenn ja, welche Art der Unterstützung wird verwendet?

- ☐ Kommunikationscomputer mit Augensteuerung
- ☐ Sprachcomputer
- ☐ Scanning-Software
- ☐ Bildschirmtastatur
- ☐ Andere: Welche Formen der elektronischen Kommunikation werden noch verwendet?

Welche Berufsgruppe ist bei der unterstützten Kommunikation federführend?

- ○ Logopädie
- ○ Neuropsychologie
- ○ Ergotherapie
- ○ Neurorehabilitationspädagogik
- ○ andere: Bitte benennen Sie die Berufsgruppe

Welche Angebote zur Angehörigenarbeit in Ihrer Einrichtung gibt es?

- ☐ Angehörigenberatung
- ☐ Angehörigengruppe
- ☐ Andere: Welche weiteren Arten von Angehörigenarbeit werden noch angeboten?

Bitte geben Sie an, welche Berufsgruppen bei der Angehörigenarbeit beteiligt sind.

- ☐ Ärzte
- ☐ Gesundheitspflegepersonal
- ☐ Psychologen
- ☐ Pädagogen
- ☐ Andere: Welche Berufsgruppen sind noch beteiligt?

Welche Facharztgruppen sind bei der Behandlung von Beatmungspatienten in Ihrer Einrichtung beteiligt?

- ☐ Anästhesiologie
- ☐ Neurologie
- ☐ Neurochirurgie
- ☐ Innere Medizin (allgemein)
- ☐ Innere Medizin und Pneumologie
- ☐ Chirurgen
- ☐ Pädiater
- ☐ Andere / Kooperation: Welche weiteren Facharztgruppen sind noch beteiligt?

- ☐ Für die Behandlung von Beatmungspatienten stehen keine Fachärzte zur Verfügung

Welche der folgenden Zusatzbezeichnungen sind bei den in Ihrer Einrichtung tätigen Ärzten vorhanden?

- ☐ Intensivmedizin: Bitte geben Sie das Gebiet / die Gebiete an (Neurologie, Anästhesiologie, etc)
- ☐ Schlafmedizin
- ☐ Palliativmedizin
- ☐ Notfallmedizin

Welches pflegerische und therapeutische Personal ist im Beatmungsbereich Ihrer Einrichtung tätig?

- ☐ Atmungstherapeuten
- ☐ Atmungstherapeuten in Weiterbildung
- ☐ Logopäden
- ☐ Physiotherapeuten
- ☐ Ergotherapeuten
- ☐ Psychologen
- ☐ Musiktherapeuten
- ☐ Physician Assistants
- ☐ Neurorehabilitationspädagogen
- ☐ Andere: Welche weiteren Berufsgruppen sind bei der Therapie noch vertreten?

Seite 19

Welches Fachgebiet ist bei der Behandlung beatmeter Patienten in Ihrer Einrichtung federführend?

- ◯ Anästhesiologie
- ◯ Neurologie
- ◯ Innere Medizin (allgemein)
- ◯ Innere Medizin und Pneumologie
- ◯ Andere/Kooperation: Welches Fachgebiet leitet die Behandlung oder wer kooperiert bei der Leitung?

- ◯ Bei der Behandlung beatmeter Patienten in unserer Einrichtung ist keine Abteilung federführend.

Seite 20

Wbldg

Welche Weiterbildungsermächtigungen für die im Beatmungsbereich tätigen Ärzte Ihrer Einrichtung liegen vor? Geben Sie bitte auch die Dauer der Ermächtigung in Monaten an.

Anästhesiologie	Dauer
Neurologie	Dauer
Innere Medizin	Dauer
Intensivmedizin (Bitte geben Sie das Gebiet / die Gebiete an):	Dauer
Evtl. noch andere Weiterbildungen?	Dauer

Seite 21

jump11

question('AD30')

1. Geben Sie bitte an, über welche Stationen Ihre Klinik verfügt.

Tragen Sie bitte den Namen der Station ein und geben Sie durch Anklicken des entsprechenden Feldes die Art der Station an. Nach jeder Eingabe klicken Sie bitte auf „Weiter".

Wenn Sie alle Stationen eingegeben haben, markieren Sie bitte das Feld „Alle Stationen wurden eingegeben" und klicken auf „Weiter".

HINWEIS: Damit durch die Angabe des Stationsnamen kein Rückschluss auf Ihre Klinik erfolgen kann, können Sie auch einen fiktiven Namen eintragen.

1 ____________

Intensivstation | IMC / Überwachung | Außerklinische Beatmung | Schlaflabor | Normalstation | andere Station

2 ____________

Intensivstation | IMC / Überwachung | Außerklinische Beatmung | Schlaflabor | Normalstation | andere Station

3 ____________

Intensivstation | IMC / Überwachung | Außerklinische Beatmung | Schlaflabor | Normalstation | andere Station

4 ____________

Intensivstation | IMC / Überwachung | Außerklinische Beatmung | Schlaflabor | Normalstation | andere Station

5 ____________

Intensivstation | IMC / Überwachung | Außerklinische Beatmung | Schlaflabor | Normalstation | andere Station

6 ____________

Intensivstation | IMC / Überwachung | Außerklinische Beatmung | Schlaflabor | Normalstation | andere Station

7 ____________

Intensivstation | IMC / Überwachung | Außerklinische Beatmung | Schlaflabor | Normalstation | andere Station

8 ____________

Intensivstation | IMC / Überwachung | Außerklinische Beatmung | Schlaflabor | Normalstation | andere Station

9 ____________

Intensivstation | IMC / Überwachung | Außerklinische Beatmung | Schlaflabor | Normalstation | andere Station

☐ Alle Stationen wurden komplett eingetragen!

Seite 23

statQuest

Seite 24

AnzBett

question('AD24')

2. Bitte geben Sie an, über wieviel Betten Ihre Stationen verfügen.

⇉ nameStation1 ⇇

⇉ nameStation2 ⇇

⇉ nameStation3 ⇇

⇉ nameStation4 ⇇

⇉ nameStation5 ⇇

⇉ nameStation6 ⇇

⇉ nameStation7 ⇇

⇉ nameStation8 ⇇

⇉ nameStation9 ⇇

Seite 25

AuswStat

question('AD26')

3. Welche Station möchten Sie bearbeiten?

Bitte wählen Sie eine Station und klicken auf WEITER.

- ◯ ⇉ nameStation1 ⇇
- ◯ ⇉ nameStation2 ⇇
- ◯ ⇉ nameStation3 ⇇
- ◯ ⇉ nameStation4 ⇇
- ◯ ⇉ nameStation5 ⇇
- ◯ ⇉ nameStation6 ⇇
- ◯ ⇉ nameStation7 ⇇
- ◯ ⇉ nameStation8 ⇇
- ◯ ⇉ nameStation9 ⇇

- ◯ Keine Station bearbeiten

Seite 26

callSub02

Seite 27

Seite 28

EndeStat

Keine Station bearbeiten

Sie haben "Keine Station bearbeiten" gewählt.

Wenn Sie den Fragebogen komplett bearbeitet haben, können Sie den Browser jetzt schließen.
Vielen Dank für Ihre Teilnahme!

Sollen die Stationen zu einem späteren Zeitpunkt bearbeitet werden, können Sie den Fragebogen über den zugesendeten Link erneut öffnen und die Befragung komplettieren.

Klicken Sie auf "WEITER", um zur Auswahl der Stationen zu gelangen.

Seite 29

Letzte Seite

Vielen Dank für Ihre Teilnahme!

Wir möchten uns ganz herzlich für Ihre Mithilfe bedanken.

Ihre Antworten wurden gespeichert, Sie können das Browser-Fenster nun schließen.
