# Supplementary material for: Weaning in early neurological-neurosurgical rehabilitation in Germany – results from a nationwide online survey
Source: Front Neurol. 2026 Jan 12;16:1700482. doi: 10.3389/fneur.2025.1700482 (PMC12832523; doi:10.3389/fneur.2025.1700482)
Supplement: Supplementary file 3 [file Data_Sheet_3.pdf]

Seite 01

Seite 02

Welche Hauptdiagnose und welche Beatmungsindikation bestehen bei diesem Patienten für den aktuellen Aufenthalt in Ihrer Einrichtung?

Erkrankung, die zum Krankenhausaufenthalt führte:	
Datum der Krankenhausaufnahme:	
Indikation zur Beatmung:	

Seite 03

Welche weiteren Diagnosen bestehen bei diesem Patienten?

- ☐ Neuromuskuläre Erkrankungen
- ☐ Neurologische Diagnosen
- ☐ Infektiologische Diagnosen
- ☐ Pneumologische Diagnose
- ☐ Kardiologische Diagnosen
- ☐ Sonstiges (Nieren- oder Leberinsuffizienz)
- ☐ Weitere Diagnosen, wenn für die Beatmungstherapie relevant: Welche anderen beatmungstherapeutisch relevanten Diagnosen wurden noch gestellt?

**Sie haben ein oder mehrere Diagnosengruppen für Ihren Patienten ausgewählt.
Bitte geben Sie die Diagnosen genauer an.**

- ☐ CIP/CIM
- ☐ Muskeldystrophie
- ☐ Guillain-Barré-Syndrom
- ☐ Myasthenie
- ☐ Amyotrophe Lateralsklerose
- ☐ Andere neuromuskuläre Erkrankung: Welche andere neuromuskuläre Erkrankung hat der Patient?
- ☐ Ischämischer Schlaganfall
- ☐ Hypoxische Enzephalopathie
- ☐ Intrakranielle Blutung
- ☐ Andere neurologische Diagnose: Welche andere neurologische Diagnose wurde gestellt?
- ☐ Sepsis
- ☐ COVID-19
- ☐ Weitere infektiologische Diagnosen: Welche Infektionen hat der Patient?
- ☐ COPD
- ☐ Andere pneumologische Diagnose: Welche andere pneumologische Erkrankung hat der Patient?
- ☐ Linksherzinsuffizienz
- ☐ Koronare Herzerkrankung
- ☐ Weitere kardiologische Erkrankungen: Welche anderen beatmungstherapeutisch relevanten Diagnosen wurden noch gestellt?
- ☐ Niereninsuffizienz, Stadium: Bitte geben Sie das Stadium an:
- ☐ Leberinsuffizienz

Ist der Patient aus einem anderen Krankenhaus zu Ihnen verlegt worden?

- ◯ Ja, der Patient wurde aus einem anderen Krankenhaus übernommen
- ◯ Nein, der Patient wurde direkt in unsere Einrichtung aufgenommen

Bitte geben Sie die folgenden Daten zum Aufenthalt in der vorbehandelnden Klinik an.

Sollten Daten Ihnen nicht bekannt sein, lassen Sie das entsprechende Feld bitte frei!

Wie lange wurde der Patient in der Vorklinik durchgehend akutstationär behandelt (Tage)? [ ]

Aus welchem vorbehandelnden Fachgebiet wurde der Patient zu Ihnen verlegt?

Wenn Ihnen dies nicht bekannt ist, geben Sie bitte im Feld „Anderes Fach“ „unbekannt“ ein.

- ○ Neurologie
- ○ Neurochirurgie
- ○ Herzchirurgie
- ○ Innere Medizin
- ○ Anderes Fach: [Welches medizinische Fachgebiet hat ihnen den Patienten überwiesen?]

Um welche Art von Klinik handelt es sich bei der verlegenden Einrichtung?

Wenn Ihnen dies nicht bekannt ist, geben Sie bitte im Feld „Anderes Haus“ „unbekannt“ ein.

- ○ Universitätsklinik
- ○ Akutkrankenhaus der Maximalversorgung
- ○ Akutkrankenhaus der Schwerpunktversorgung
- ○ Akutkrankenhaus der Grundversorgung
- ○ Anderes Haus: [Aus welcher Einrichtung kam ihr Patient?]

Auf welcher Station wurde der Patient in der vorbehandelnden Einrichtung versorgt?

Wenn Ihnen dies nicht bekannt ist, geben Sie bitte im Feld „Andere Station“ „unbekannt“ ein.

- ○ Intensivstation
- ○ IMC
- ○ Stroke Unit
- ○ Andere Station: [Von welcher Station wurde ihr Patient verlegt?]

Welches Geschlecht hat der Patient?

- ○ weiblich
- ○ männlich
- ○ divers

Bitte geben Sie die folgenden Patientendaten ein.

Größe (cm):

Gewicht (kg):

Alter (Jahre):

Bitte geben Sie an, wie viele Tage insgesamt und wie viele Stunden innerhalb der letzten 24h der Patient in Ihrer Einrichtung beatmet wurde.

Beatmungstage insgesamt:

Beatmungsstunden innerhalb der letzten 24h:

Beatmungsstunden in der aktuellen Einrichtung insgesamt

Sind Therapiebegrenzungen bei Ihrem Patienten vereinbart?
Wenn ja, welche?

- ☐ Keine kardiopulmonale Reanimation
- ☐ Keine Beatmung
- ☐ Keine Therapie auf der Intensivstation
- ☐ Keine Katecholamine/Inotropika
- ☐ Keine Nierenersatztherapie
- ☐ Keine Antibiose
- ☐ Keine enterale oder parenterale Ernährung
- ☐ Ein Verzicht auf Therapieeskalation wurde vereinbart („Therapie-Freezing“)
- ☐ Lebenserhaltender Therapien wie Beatmung, Ernährung oder Flüssigkeitsgabe sind eingestellt worden
- ☐ Andere: Welche andere Therapiebegrenzung wurde festgelegt?

- ☐ Es gibt keine Therapiebegrenzungen

Seite 10

Auf welcher Rechtsgrundlage beruht die Therapiebegrenzung / Therapiebeendigung?

- ◯ Mündlich geäußerter Patientenwille
- ◯ Patientenverfügung
- ◯ Patientenwille ermittelt auf der Basis von Angaben des gesetzlichen Vertreters
- ◯ Medizinische Indikation (z.B. bei infauster Prognose, malignes Grundleiden o.ä.)
- ◯ Sterbephase hat begonnen

Seite 11

jump7

Sind bei Ihrem Patienten Isolationsmaßnahmen ergriffen worden?

- ☐ JA, der Patient ist isoliert worden

Seite 12

jump8

Sie haben angegeben, dass Sie Ihren Patienten isoliert haben. Bitte geben Sie den Grund für die Isolationsmaßnahmen an.

- ☐ Prophylaktisch bei Aufnahme / Übernahme
- ☐ 3-MRGN
- ☐ 4-MRGN
- ☐ VRE
- ☐ COVID-19
- ☐ Sonstige: Bitte geben Sie den Grund der Isolationsmaßnahme an

---

Seite 13

jump6

Seite 14

question('PD17', '1-9')

Welche Untersuchungsverfahren wurden während des gesamten aktuellen stationären Aufenthaltes in Ihrer Einrichtung durchgeführt?

- ☐ Transkutane Kapnometrie
- ☐ Blutgasanalyse
- ☐ Spirometrie
- ☐ Polygraphie
- ☐ Bronchoskopie
- ☐ Fiberendoskopische Evaluation des Schluckens (FEES)
- ☐ cMRT
- ☐ cCT
- ☐ Andere CT- / MRT-Untersuchungen
  Bitte geben Sie die Art der Untersuchung an

Seite 15

question('PD17', '10-19')

Welche Untersuchungsverfahren wurden während des gesamten aktuellen stationären Aufenthaltes in Ihrer Einrichtung durchgeführt?

- ☐ EMG/NLG
- ☐ Evozierte Potentiale
- ☐ EEG
- ☐ Liquordiagnostik
- ☐ Andere: Welche weiteren Untersuchungen wurden noch durchgeführt?

Bitte machen Sie Angaben zur Beatmungssituation Ihres Patienten.

Art des Beatmungsgerätes		[Bitte auswählen]
Bitte geben Sie den Beatmungsmodus an!		[Bitte auswählen]
Bitte geben Sie den letzten eingestellten PEEP an (cm H2O).	0	70 cm H2O
Letzte voreingestellte FiO2 (Prozent)	21 %	100 %

Über welchen Beatmungszugang findet die Beatmung Ihres Patienten statt?

- ☐ Tubus (nasotracheal, orotracheal)
- ☐ Trachealkanüle
- ☐ Sonstige
  Bitte geben Sie die Art des Beatmungszugangs an

Welche Patientenparameter wurden in den letzten 24h überwacht?

- ☐ EKG
- ☐ NiBP
- ☐ SaO2
- ☐ Temperatur
- ☐ invasiver Blutdruck
- ☐ etCO2
- ☐ BGA
- ☐ Dauer-EEG
- ☐ ICP
- ☐ HZV-Monitoring (z.B. PiCCO, Pulmonaliskatheter o.ä.)

Seite 19

Hat der Patient eines oder mehrere der folgenden Verfahren innerhalb der letzten 24 Stunden benötigt?

- ☐ Mechanischer Insufflator-Exsufflator (Hustenassistent)
- ☐ Kontinuierliche Nierenersatzverfahren
- ☐ Intermittierende Nierenersatzverfahren
- ☐ Left Ventricular Assist Device (LVAD)

- ☐ Keins der Verfahren ist innerhalb der letzten 24 Stunden benötigt worden.

Seite 20

Mit welchen der folgenden Drainagen, Sonden und Kathetern ist Ihr Patient derzeit versorgt?

- ☐ Thoraxdrainage
- ☐ Zentraler Venenkatheter
- ☐ Nasale Magensonde
- ☐ Perkutane, endoskopische Gastrostomie (PEG)/ oder -Jejunustomie (PEJ)
- ☐ Blasendauerkatheter
- ☐ Suprapubischer Blasenkatheter
- ☐ Ventrikeldrainage
- ☐ Lumbaldrainage

Seite 21

lastside

Letzte Seite

Vielen Dank für Ihre Teilnahme!

Wir möchten uns ganz herzlich für Ihre Mithilfe bedanken.

Ihre Antworten wurden gespeichert, Sie können das Browser-Fenster nun schließen.
