# Supplementary material for: Weaning in early neurological-neurosurgical rehabilitation in Germany – results from a nationwide online survey
Source: Front Neurol. 2026 Jan 12;16:1700482. doi: 10.3389/fneur.2025.1700482 (PMC12832523; doi:10.3389/fneur.2025.1700482)
Supplement: Supplementary file 4 [file Data_Sheet_4.pdf]

Seite 01

Seite 02

Start Station

Eingabe der Stationsdaten

Bevor Sie mit dem Beantworten der Fragen beginnen, die Ihre Patienten betreffen, möchten wir Sie bitten, die Fragen zu Ihrer Station zu beantworten.

Diese Fragen müssen nur einmal beantwortet werden.

Seite 03

Sie haben für die Station ⇉ stat ⇇ eine Bettenanzahl von ⇉ anz ⇇ angegeben.
Bitte geben Sie an, wieviel der Betten für Intensiv- und Beatmungspatienten vorgesehen sind.

Anzahl der Intensivbetten

Anzahl der Beatmungsbetten

Sollten Sie feststellen, dass die Gesamtanzahl der Betten falsch angegen wurde, haben Sie die Möglichkeit, diese zu korrigieren.

Wenn Sie eine Korrektur der Bettenanzahl vornehmen möchten, setzen Sie bitte ein Häkchen durch Anklicken des Auswahlfeldes und dann auf WEITER.

☐ Korrektur der Anzahl der Gesamtbetten vornehmen

Seite 04

Seite 05

Seite 06

question('SD28', '1-')

1. Bitte geben Sie an, auf welchen Bettplätzen beatmete Patienten liegen.

- ☐ Bettplatz 1

Seite 07

Seite 08

2. Welche der folgenden Operationen- und Prozedurenschlüssel (OPS) werden für die Patienten kodiert, die auf Ihrer Station behandelt werden?

- ☐ 1-717: Feststellung des Beatmungsstatus und des Beatmungsentwöhnungspotenzials
- ☐ 8-552: Neurologisch-neurochirurgische Frührehabilitation
- ☐ 8-718.8: Prolongierte Beatmungsentwöhnung auf spezialisierter intensivmedizinischer Beatmungsentwöhnungs-Einheit
- ☐ 8-718.9: Prolongierte Beatmungsentwöhnung auf spezialisierter nicht intensivmedizinischer Beatmungsentwöhnungs-Einheit
- ☐ 8-980: Intensivmedizinische Komplexbehandlung (Basisprozedur)
- ☐ 8-98f: aufwendige intensivmedizinische Komplexbehandlung

- ☐ Keine der aufgeführten Operationen- und Prozedurenschlüssel (OPS) werden kodiert

Seite 09

Bitte beschreiben Sie kurz Ihre Patientenkonferenzen.
Wie häufig finden diese in der Woche statt?

	täglich	sechsmal	fünfmal	viermal	dreimal	zweimal	einmal	überhaupt nicht
Häufigkeiten der Konferenzen pro Woche	○	○	○	○	○	○	○	○

Bitte beschreiben Sie kurz Ihre Patientenkonferenzen. Wie häufig und wie lange finden diese statt, wie viele Patienten werden im Mittel besprochen, wie werden die Konferenzen dokumentiert, werden spezielle Themen wie z.B. Ethik, Palliative Care, Atmungstherapie oder Weaning behandelt?

- ☐ Besprechung der Themen Ethik/Palliativ Care
- ☐ Besprechung der Themen Atmungstherapie/Weaning

question('SD19', 'combine=SD20', 'combine=SD21', 'combine=SD22')

Welche Arztgruppen nehmen an den Patientenkonferenzen teil und welchen Status haben diese?

	Chefarzt	Oberarzt	Facharzt	Assistenzarzt
Anästhesie	☐	☐	☐	☐
Neurologie	☐	☐	☐	☐
Innere Medizin	☐	☐	☐	☐
Andere: Bitte Fachrichtung und Status angeben.	☐	☐	☐	☐

Welche weiteren Berufsgruppen nehmen an den Patientenkonferenzen teil?

- ☐ Gesundheits-/Krankenpflegekraft
- ☐ Atmungstherapeuten
- ☐ Atmungstherapeuten in Weiterbildung
- ☐ Logopäden
- ☐ Physiotherapeuten
- ☐ Ergotherapeuten
- ☐ Psychologen
- ☐ Neuropsychologen
- ☐ Musiktherapeuten
- ☐ Kunsttherapeuten
- ☐ Physician Assistants
- ☐ Neurorehabilitationspädagogen
- ☐ Andere: Welche Berufsgruppen nehmen sonst noch teil?

question('SD23', 'combine=SD24', 'combine=SD25', 'combine=SD26')

Welche Arztgruppen nehmen an den Visiten teil und welchen Status haben diese?

	Chefarzt	Oberarzt	Facharzt	Assistenzarzt
Anästhesie	☐	☐	☐	☐
Neurologie	☐	☐	☐	☐
Innere Medizin	☐	☐	☐	☐
Andere: Bitte Fachrichtung und Status angeben.	☐	☐	☐	☐

Bitte beschreiben Sie kurz Ihre Visiten. Wie lang ist in der Regel die Gesamtdauer der Visite und wie viele Patienten werden dabei im Mittel besprochen? Wie erfolgt die Dokumentation der Visite?

Bitte geben sie die Patientenzahl nur bei der für sie (in der Regel) zutreffenden Gesamtdauer der Visite an!

- ☐ Dokumentation der Visite elektronisch
- ☐ Dokumentation der Visite auf Papier

Welche weiteren Berufsgruppen nehmen an den Visiten teil?

- ☐ Gesundheits-/Krankenpflegekraft
- ☐ Atmungstherapeuten
- ☐ Atmungstherapeuten in Weiterbildung
- ☐ Logopäden
- ☐ Physiotherapeuten
- ☐ Ergotherapeuten
- ☐ Psychologen
- ☐ Neuropsychologen
- ☐ Musiktherapeuten
- ☐ Kunsttherapeuten
- ☐ Physician Assistants
- ☐ Neurorehabilitationspädagogen
- ☐ Andere: Welche weiteren Berufsgruppen nehmen noch an der Visite teil?

DIVI-Kriterien

Sie geben Daten für Ihre Intensivstation ein.

Bitte beantworten Sie noch die folgenden Fragen zu den DIVI-Kriterien.

Bitte geben Sie an ob folgende DIVI-Kriterien, das Personal betreffend, für Ihre Einrichtung zutreffen.

- ☐ Leitung durch Arzt mit Zusatzbezeichnung „Intensivmedizin“ und hauptamtlicher Tätigkeit auf der Intensivstation
- ☐ Ausfallkompensation für den ärztlichen Leiter
- ☐ 24h-Präsenz des ärztlichen Dienstes auf der Intensivstation
- ☐ 24-h-Präsenz eines Arztes mit der Weiterbildung „Intensivmedizin“ in der Einrichtung
- ☐ Sieben Arztstellen für 8 bis 12 Betten
- ☐ 1 Pflegekraft für 2 Behandlungsplätze pro Schicht (Frühschicht und Spätschicht)
- ☐ Anteil an qualifizierten Intensiv-Fachpflegekräften mindestens 30 % des Pflegeteams der Intensivstation
- ☐ Teilnahme eines klinischen Pharmakologen/Apothekers/Pharmazeuten mindestens einmal wöchentlich an der Visite
- ☐ Verfügbarkeit eines klinischen Pharmakologen/Apothekers/Pharmazeuten jederzeit gegeben
- ☐ Seelsorgerische Betreuung kurzfristig verfügbar
- ☐ Permanente Präsenz von Anästhesiologie auf Facharztniveau im Haus
- ☐ Permanente Präsenz von Allgemein-/Viszeralchirurgie auf Facharztniveau im Haus
- ☐ Permanente Präsenz von Innerer Medizin auf Facharztniveau im Haus
- ☐ Kurzfristige Verfügbarkeit von Neurologie auf Facharztniveau
- ☐ Kurzfristige Verfügbarkeit von TEE auf Facharztniveau
- ☐ Kurzfristige Verfügbarkeit von Koronarangiographie auf Facharztniveau
- ☐ Kurzfristige Verfügbarkeit von Notfallendoskopie auf Facharztniveau
- ☐ Kurzfristige Verfügbarkeit von Urologie auf Facharztniveau

Seite 18

Bitte geben Sie an ob folgende DIVI-Kriterien, das Personal betreffend, für Ihre Einrichtung zutreffen.

- [ ] Lokalisation in der Nähe von OP-Einheit, Aufwachraum, Notaufnahme, Koronarangiographie, Radiologie und Intermediate Care Station
- [ ] Zimmergröße mindestens 25 m² bei Einzelzimmern, mindestens 40 m² bei Zweibettzimmern.
- [ ] Einzelzimmer generell Schleuse oder ein Isolierzimmer mit Vorraum (2 m²) pro 6 Betten
- [ ] Eingriffsraum vorhanden
- [ ] 1-2 Geräteräume (ca. 25 m²) mit Arbeitstischanlage vorhanden
- [ ] Behindertengerechte kombinierte Toiletten-, Wasch- und Duscheinheit vorhanden
- [ ] Oberarztzimmer vorhanden
- [ ] Stationsleitungszimmer vorhanden
- [ ] Physiotherapie-Arbeitsraum vorhanden

Seite 19

Bitte geben Sie an ob folgende DIVI-Kriterien, die Infrastruktur betreffend, für Ihre Einrichtung zutreffen.

- [ ] Zentrallabor mit 24-stündiger Besetzung im Haus
- [ ] Cito-Labor im Stationsbereich (ca. 10 m²)
- [ ] Permanente Verfügbarkeit von Radiologie einschließlich CT und mobilem Röntgengerät
- [ ] Permanente Verfügbarkeit von Blutprodukten
- [ ] Tägliche Verfügbarkeit eines mikrobiologischen Labors
- [ ] Tägliche Verfügbarkeit des MRT
- [ ] PDMS

Seite 20

Bitte geben Sie an ob folgende DIVI-Kriterien, das Monitoring betreffend, für Ihre Einrichtung zutreffen.

.

- [ ] Möglichkeit des Monitorings von folgenden Parametern: EKG, arteriellem und zentralvenösem Druck, Pulsoxymetrie, endtidaler Kapnometrie,Temperatur (2 Kanäle), hämodynamische Überwachung (ST-Segmentüberwachung und ≥2 Verfahren des invasiven hämodynamischen Monitorings wie HZV, ScvO2, Widerstände, Füllungsdrücke oder Volumina) (ALLE Verfahren müssen möglich sein!)
- [ ] Möglichkeit der Kapnometrie an allen Beatmungsplätzen
- [ ] Monitoring mit gleichzeitiger Anzeige von ≥1 EKG-Ableitungen, ≥2 invasiven Drucken (arterieller Druck, zentralvenöser Druck), SpO2 (numerische Werte und Kurven), nicht-invasivem Blutduck und Temperatur
- [ ] EEG- oder CFM-Monitoring
- [ ] Beatmungsgeräte mit Überwachungsfunktionen
- [ ] Möglichkeit der nicht-invasiven Beatmung

question('DI05', '1-16')

Bitte markieren Sie die in Ihrer Einrichtung vorhandenen Geräte.

- [ ] Blutgasanalysegerät
- [ ] Point-of-Care Messung von Na, K, Ca, Hb und Laktat (alle Parameter !)
- [ ] Point-of-Care-Messung von Glukose
- [ ] Point-of-Care-Messung der Gerinnung (z.B. INR, PTT, ACT)
- [ ] Thrombelastographie (z.B. Rotem)
- [ ] Impedanz-Aggregometrie (z. B. Multiplate)
- [ ] Bronchoskop
- [ ] Ultraschallgerät
- [ ] Sektorschallkopf Abdomen
- [ ] Sektorschallkopf Kardio
- [ ] Linearschallkopf
- [ ] TEE-Sonde
- [ ] Duplex
- [ ] Doppler
- [ ] CVVHD und Intermittierende Nierenersatzverfahren
- [ ] Intrakranielle Druckmessung

question('DI05', '17-33')

Bitte markieren Sie die in Ihrer Einrichtung vorhandenen Geräte.

- ☐ Infusions- und Perfusorpumpen an allen Intensivbetten
- ☐ Relaxometrie
- ☐ Druckmanschetten für Druckinfusion
- ☐ Blutwärmer und GFP-Auftaugerät
- ☐ Schnelltransfusionsgerät
- ☐ Externe Kühlungsvorrichtung für Patienten
- ☐ Wärmedecken
- ☐ Invasive Kühlung/Erwärmung
- ☐ Spezialmatratzen
- ☐ Mehrkanal-EKG
- ☐ Defibrillator (≥2) mit externem Herzschrittmacher
- ☐ Transvenöser Schrittmacher (Katheter, SM-Aggregat)
- ☐ Transportmonitore, Anzahl: Bitte geben sie die Anzahl der vorhandenen Transportmonitore an.
- ☐ Transportrespirator
- ☐ Waage
- ☐ IABP (intraaortale Ballongegenpulsation)
- ☐ ECMO, ECLA oder ILA

statEnd

question('SD12', 'show-title=no', 'spacing=2')

Wählen Sie einen Bettenplatz aus und klicken Sie auf WEITER.

- ◯ Bettplatz1 ⇉ bearb1 ⇇
- ◯ Bettplatz2 ⇉ bearb2 ⇇
- ◯ Bettplatz3 ⇉ bearb3 ⇇
- ◯ Bettplatz4 ⇉ bearb4 ⇇
- ◯ Bettplatz5 ⇉ bearb5 ⇇
- ◯ Bettplatz6 ⇉ bearb6 ⇇
- ◯ Bettplatz7 ⇉ bearb7 ⇇
- ◯ Bettplatz8 ⇉ bearb8 ⇇
- ◯ Bettplatz9 ⇉ bearb9 ⇇
- ◯ Bettplatz10 ⇉ bearb10 ⇇
- ◯ Bettplatz11 ⇉ bearb11 ⇇
- ◯ Bettplatz12 ⇉ bearb12 ⇇
- ◯ Bettplatz13 ⇉ bearb13 ⇇
- ◯ Bettplatz14 ⇉ bearb14 ⇇
- ◯ Bettplatz15 ⇉ bearb15 ⇇
- ◯ Bettplatz16 ⇉ bearb16 ⇇
- ◯ Bettplatz17 ⇉ bearb17 ⇇
- ◯ Bettplatz18 ⇉ bearb18 ⇇
- ◯ Bettplatz19 ⇉ bearb19 ⇇
- ◯ Bettplatz20 ⇉ bearb20 ⇇
- ◯ Bettplatz21 ⇉ bearb21 ⇇
- ◯ Bettplatz22 ⇉ bearb22 ⇇
- ◯ Bettplatz23 ⇉ bearb23 ⇇
- ◯ Bettplatz24 ⇉ bearb24 ⇇
- ◯ Bettplatz25 ⇉ bearb25 ⇇
- ◯ Bettplatz26 ⇉ bearb26 ⇇
- ◯ Bettplatz27 ⇉ bearb27 ⇇
- ◯ Bettplatz28 ⇉ bearb28 ⇇
- ◯ Bettplatz29 ⇉ bearb29 ⇇
- ◯ Bettplatz30 ⇉ bearb30 ⇇
- ◯ Bettplatz31 ⇉ bearb31 ⇇
- ◯ Bettplatz32 ⇉ bearb32 ⇇
- ◯ Bettplatz33 ⇉ bearb33 ⇇
- ◯ Bettplatz34 ⇉ bearb34 ⇇
- ◯ Bettplatz35 ⇉ bearb35 ⇇
- ◯ Bettplatz36 ⇉ bearb36 ⇇
- ◯ Bettplatz37 ⇉ bearb37 ⇇
- ◯ Bettplatz38 ⇉ bearb38 ⇇
- ◯ Bettplatz39 ⇉ bearb39 ⇇
- ◯ Bettplatz40 ⇉ bearb40 ⇇
- ◯ Bettplatz41 ⇉ bearb41 ⇇
- ◯ Bettplatz42 ⇉ bearb42 ⇇
- ◯ Bettplatz43 ⇉ bearb43 ⇇

- ◯ Bettplatz44 ⇉ bearb44 ⇇
- ◯ Bettplatz45 ⇉ bearb45 ⇇
- ◯ Bettplatz46 ⇉ bearb46 ⇇
- ◯ Bettplatz47 ⇉ bearb47 ⇇
- ◯ Bettplatz48 ⇉ bearb48 ⇇
- ◯ Bettplatz49 ⇉ bearb49 ⇇
- ◯ Bettplatz50 ⇉ bearb50 ⇇

- ◯ Keine weiteren Bettplätze bearbeiten – Zurück zur Stationsauswahl

---

Seite 25

subStart

Seite 26

subRep

Seite 27

sub02End

Seite 28

Letzte Seite

Vielen Dank für Ihre Teilnahme!

Wir möchten uns ganz herzlich für Ihre Mithilfe bedanken.

Ihre Antworten wurden gespeichert, Sie können das Browser-Fenster nun schließen.
